# Characterization of resistant MCF-7 breast cancer cells developed by repeated cycles of photodynamic therapy

**DOI:** 10.3389/fphar.2022.964141

**Published:** 2022-09-16

**Authors:** Eric Chekwube Aniogo, Blassan P. George, Heidi Abrahamse

**Affiliations:** Laser Research Centre, Faculty of Health Sciences, University of Johannesburg, Doornfontein, South Africa

**Keywords:** breast cancer, photodynamic therapy, p-glycoprotein, MCF-7 cells, multidrug resistance

## Abstract

Breast cancer mainly affects women and causes a severe global threat to health. It is often managed and treated with surgery, chemotherapy, immunotherapy, and radiation therapy. Generally, chemotherapy as a treatment option is often opposed by responsive tumor relapse and development of resistance, a significant setback of current treatment. Photodynamic therapy (PDT) offers a promising modality that can treat cancer by combining a photosensitizer and laser irradiation in the presence of oxygen. However, one problem of PDT in treating breast cancer is the apparition of the resistant cell population. Thus, we aimed for stepwise selection and characterization of MCF-7 cells resistant to PDT with a sulfonated zinc phthalocyanine (ZnPcS4) photosensitizer. The wild-type MCF-7 was exposed to successive cycles of ZnPcS4-PDT, and 10resistant populations were finally obtained. In wild-type and parental cells, we analyzed the cell morphology (light microscopy), cell cycle (BrdU staining), cell viability (MTT assay), antioxidant activity (superoxide dismutase measurement), and immunofluorescence expression of resistant p-glycoprotein (P-gp). The results indicate that resistant cells showed a mesenchymal cell phenotype, few differences in antioxidant activity, an increased DNA synthesis, and more expression of P-gp than the wild-type parental cells. These distinctive features of resistant cells can provide insight into the emergence of MCF-7 cell resistance to PDT, which was necessary to design the best therapeutic procedure for improved efficacy.

## 1 Introduction

Breast cancer is the most frequently occurring cancer in women, with an estimated incidence of 2.3 million new cases in 2020 (Sung et al., 2021). According to the latest 2020 statistics, breast cancer is the fifth leading cause of cancer mortality worldwide, primarily seen in developing countries of Melanesia, the Caribbean, and sub-Saharan Africa despite the 88% higher incidence rate found in developed countries of Australia, New Zealand, and Western Europe (Sung et al., 2021). Standard treatment procedures for breast cancer include surgery, chemotherapy, immunotherapy, and radiation therapy (Aniogo et al., 2017). Over the past decades, cancer chemotherapy, radiation, and targeted therapies have advanced in patient management and have led to decreased mortality; this tremendous progress is not as expected because the disease seems not to be extinguished (Barrera-Rodríguez and Fuentes, 2015). Photodynamic therapy is an emerging treatment regime that systematically uses a light-sensitive chemical called a photosensitizer (PS) in light and oxygen to induce the destruction of cancerous cells (Allison and Moghissi, 2013). The laser system has fiber optics that helps the accurate delivery of light sufficient to activate the photosensitizer located inside the tumor cells. Photosensitizers absorb light of a specific wavelength between 600–800 nm, providing enough energy to generate oxygen radicals that will trigger cell death (Agostinis et al., 2004). PDT and other cancer treatments such as cancer vaccines and immunotherapy are currently under increased consideration in preclinical studies for use in breast cancer treatment. Experimental data have shown PDT to be a promising treatment option with reduced long-term mobility, limited side effect, and better cancer specificity over surgery, chemotherapy, or radiotherapy (Ostańska et al., 2021). The efficiency of PDT treatment depends on the production of reactive oxygen species (ROS) that initiate membrane damage and cellular death. ROS directly kills tumor cells and induces inflammatory and immune responses with tumor vasculature shutdown that effectively leads to tumor control (Postiglione et al., 2011). Cells may survive PDT due to poor photosensitizer localization, inadequate illumination, and reduced generation of ROS. In this case, the surviving cells may have higher resistance and malignancy than the initial parental cells. The inability to suffer death after PDT treatment means a selective advantage in tumor progression (Vilchez et al., 2021). Exposure to repeated cycles of PDT is a robust selection process that allows only the most resistant cells to survive (Ghahe et al., 2021; Vilchez et al., 2021). Research has shown that repetitive ALA-mediated PDT can isolate resistant murine adenocarcinoma for the mechanistic setting of new, potential targets and the development of cancer resistance novel therapeutic strategies (Casas et al., 2006). This has increased the need for more research on other cell lines to elucidate the drivers of resistance after PDT in MCF-7 cells.

Multidrug resistance (MDR) is a mechanism in which tumor cells become insensitive to the cytotoxic effects of structural and functionally unrelated drugs used for cancer treatment (Wu et al., 2014). It is a phenomenon that involves a multitude of contributing factors that determine either intrinsic or acquired resistance to cancer (Videira et al., 2014). PDT is an emerging treatment option with admirable efficacy in breast cancer treatment. However, breast cancer-induced development of resistance has become a significant challenge to its successful integration into the clinics. It is estimated that up to half of breast cancer patients are not responsive to current regimens (O’driscoll and Clynes, 2006). The development of pleiotropic resistance has been linked to membrane proteins, such as members of ATP-binding cassette (ABC) transporter proteins, including p-glycoprotein (P-gp), breast cancer resistance proteins, and multi-drug resistance-associated proteins which are thought to pump drugs out of the cytoplasm (Boichuk et al., 2017). These efflux transporters play a vital role in protecting cells in physiological conditions, by pumping out metabolites and their conjugates (Saxena et al., 2011). P-gp plays a vital role in developing multidrug resistance in breast cancer cells. Like in many tumors, its increased expression can lead to an efflux of compounds like PS, which reduces the efficacy of PDT (Ge et al., 2017). Both experimental and clinical data have linked the sensitivity of cancer treatment and patients’ survival to negatively correlate with P-gp expression (Amirzada et al., 2014). MDR has been associated with cells surviving under chemotherapy, highlighting the importance of the innovative development of new therapeutic strategies to overcome cancer resistance. PDT has been proposed as an alternative approach. Hence, it is essential to study the development of its resistance mechanism. Because PDT can be repeated multiple times with no cumulative toxic effects, its usefulness can be utilized to define acquired resistance development through its repetitive cycles, which provides an insight into the relative merits of invasive tumor prevention and possible therapeutic target. The objective of the current research was to isolate and characterize MCF-7 cells resistant to repeated photodynamic treatments with sulfonated zinc phthalocyanine. The characteristics of the resistant cells could provide insight to facilitate understanding the emergence of MCF-7 cell resistance to design the best therapeutic procedure with improved efficacy.

## 2 Materials and methods

### 2.1 Cell line and culture conditions

The MCF-7 cell line obtained from the American Type Culture Collection (ATCC: HTB 22) was used in the study as wild-type (WT) parental cells. The cells were maintained in a tissue culture flask with Dulbecco’s Modified Eagle’s Medium (DMEM) (D5796), supplemented with 10% fetal bovine serum (FBS, Gibco, 306.00301), 1% amphotericin B (A2942), and 1% penicillin–streptomycin (Sigma, P4333). Incubation was performed at 85% humidified condition at 37°C and 5% CO_2._ The cells were grown in a T75 culture flask (CR/431080), subcultured, and washed with HBSS (H9394) twice a week when confluent and detached from the culture flask by single-cell suspension formation using TrypLE^tm^ Express (Gibco, 12,604). Experimental cells were seeded at 5 × 10^5^ cells/mL concentration in 35 mm culture dishes and incubated for 4 h to attach, homeostatic recovery before treatment.

### 2.2 Induction of resistance and isolation of MCF-7/photodynamic therapy-resistant cells

Commercially purchased zinc tetrasulfonic acid phthalocyanine (ZnPcS4) from Santa Cruz® Biotechnology (sc-264509A), dissolved in phosphate-buffered saline (PBS), was used as a photosensitizer (PS) which absorbs light at 674 nm. The PS at 20 µm concentration and 20 J/cm^2^ fluence was previously reported to inhibit a 50% decrease (IC50) in MCF-7 cells (Chekwube et al., 2020) and, hence, was used as a benchmark in the present study. Wild-type (WT) MCF-7 cells were incubated at 5 × 10^5^ cells/mL concentration in 35 mm culture dishes, and 20 μm PS was added and incubated overnight before PDT laser irradiation. A diode laser of 680 nm wavelength was used for irradiation, and the cells were irradiated in the dark without lids at 20 J/cm^2^ fluence. The duration of the laser exposure was calculated as follows: irradiance (J/cm^2^) = time (s) × {power (W)/surface (cm^2^)) (Yang et al., 2011). The surviving cells were cultured again with fresh media, and the cells were allowed to proliferate to 90–100% confluence before another PDT cycle was repeated. After ten (10) repetitive cycles of the PDT over 62 days, the surviving cells were termed PDT-resistant MCF-7 (MCF-7/PDT) cells. Untreated control (WT MCF-7 cells) was also cultured in a complete culture medium under similar conditions of repetitive cycles.

### 2.3 Characterization of MCF-7/photodynamic therapy cells resistant to ZnPcS4-PDT

#### 2.3.1 Examination of morphological changes in cells

The inverted light microscope (Olympus, CKX41) was used to visualize the morphological changes of both WT and MCF-7/PDT cells. Irregular cell membrane, rounding up, and floating cells in the culture medium were examined.

#### 2.3.2 Flow cytometry

Cells were seeded in Petri dishes (35 mm) at 5 × 10^5^ cells/mL concentration, detached with TrypLE, and transferred in flow cytometric tubes. After that, they were stained with monoclonal mouse P-gp antibody (MA5-13854) and incubated for 4 h. Subsequently, the cells were counterstained with FITC goat anti-mouse (NovusBio, NB720) for 1 h and washed with PBS by centrifuging at 400xg for 5 min and the pellet resuspended in 500 µL of ice-cold PBS. The suspension was measured for fluorescence with a BD Accuri flow cytometer.

#### 2.3.3 Immunofluorescence

Immunofluorescence microscopy was performed to detect resistant proteins like the p-glycoprotein (P-gp) qualitatively. Briefly, cells were grown on a coverslip, fixed with 4% paraformaldehyde, permeabilized with 0.1% Triton X-100 in PBS, blocked for non-specific binding with 10% mouse serum in PBS, and exposed to an unlabeled primary anti-P-gp antibody. The slides were washed and incubated for 1 h at room temperature with a secondary fluorescence-labeled antibody that binds to the primary antibody. The cell nuclei were counterstained with 1 μg/ml 4′,6-diamidino-2-phenylindole (DAPI). The coverslip was inverted onto a glass slide, mounted, and observed under a Carl Zeiss Axio Observer ZI fluorescence microscope.

### 2.4 Measurement of superoxide dismutase activity

This assay measures the levels of antioxidant activities among the two variant cells. High levels of antioxidants prevent/repair the damage caused by ROS and, thus, can lead to cell resistance to PDT. The assay was performed according to the SOD activity assay kit (CS0009) protocol. In brief, MCF-7/PDT cells were lysed, and the lysate was centrifuged at 4°C (14 000xg for 5 min). After that, 20 µL of the supernatant containing SOD was transferred to a 96-well culture plate. The reaction was initiated by adding 20 µl of xanthine oxidase to each well, and the mixture was incubated at 20–25°C for 30 min. The absorbance was detected by using a spectrophotometer at 450 nm (Perkin-Elmer VICTOR™).

### 2.5 Cell growth analysis

The MCF-7/PDT cells in exponential growth were harvested with TrypLE and resuspended culture media before the assessment of cellular viability using a 3-(4,5-dimethylthiazol-2-yl)-2,5-diphenyltetrazolium bromide (MTT) (Sigma-Aldrich) colorimetric assay. This assay is based on reducing MTT tetrazole salt to a purple formazan that occurs in viable cell mitochondria. The measurement of formazan dye is proportional to the viability of the cell.

### 2.6 Cell cycle analysis

This analysis will determine different cycle stages of MCF-7/PDT cells treated with repetitive PDT. Cells were suspended at 5×10^4^ cells per 200 µl and washed with PBS. After that, cells were mixed with 2 ml of ice-cold 70% ethanol and fixed on ice for 30 min. The suspension was centrifuged for 5 min at 400 x g, and the supernatant was discarded. Further cell suspension was performed with 400 µl of PBS, 50 µl of RNAse (25 mg/ml), and 10 µl of propidium iodide (0.5 mg/ml). Samples were analyzed on a BD Accuri C6 flow cytometer.

### 2.7 Statistical analysis

The experiments were repeated four times (*n* = 4), and a one-way ANOVA statistical analysis was used to determine the significance between controls (WT-MCF-7 untreated) and MCF-7/PDT-treated samples. Data represented the mean ± standard error of the mean (SEM). The statistical significance was analyzed using SigmaPlot version 14.0. and were presented as *p < 0.05, **p < 0.01, and ***p < 0.001.

## 3 Results

### 3.1 Induction of resistance to ZnPcS4-PDT

**FIGURE 1:**
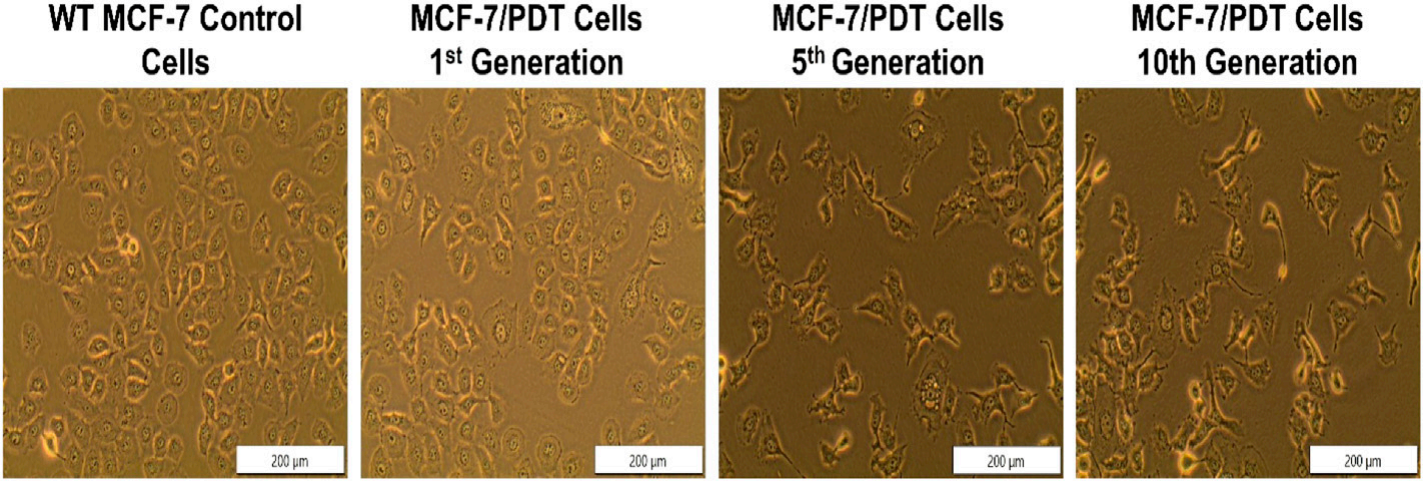
Morphological assessment of WT-MCF-7 cells and different generations of MCF-7/PDT cells. There were no observable changes except the transition from epithelial to mesenchymal cell phenotype after repeated cycles of PDT (magnification 200 µm).

To develop MCF-7/PDT cells resistant to 10 repetitive cycles of PDT, wild-type MCF-7 cells were incubated with ZnPcS4 overnight and later exposed to laser irradiation at 20 J/cm^2^ fluence. The PDT treatment condition caused a 50% survival rate inhibition in WT MCF-7 cells. The surviving cells were replenished with a complete medium until they increased to 90–100% confluence, and the PDT treatment cycle was repeated 10 times under similar conditions. The PDT-resistant cells were also taken at some specific generation during the repetitive PDT cycles at 1st, 5th, and 10th cycles and were designated as 1st, 5th, and 10th generation cells. This is to observe the tolerance and stability of the cells to PDT and to study the biological characteristics of the resistant population by comparing it to WT MCF-7 cells. The last cells that survived the 10 cycles of PDT were designated as MCF-7/PDT cells. Figure 1 shows the morphological assessment of the WT MCF-7 cells, 1st, 5th, and 10th generations. The WT cells showed no observable changes, but the 5th and 10th generations showed a fibroblastic nature of cells which seems to have transitioned from epithelial to mesenchymal cell phenotype (Figure 1).

### 3.2 Measurement of P-glycoprotein expression

**FIGURE 2:**
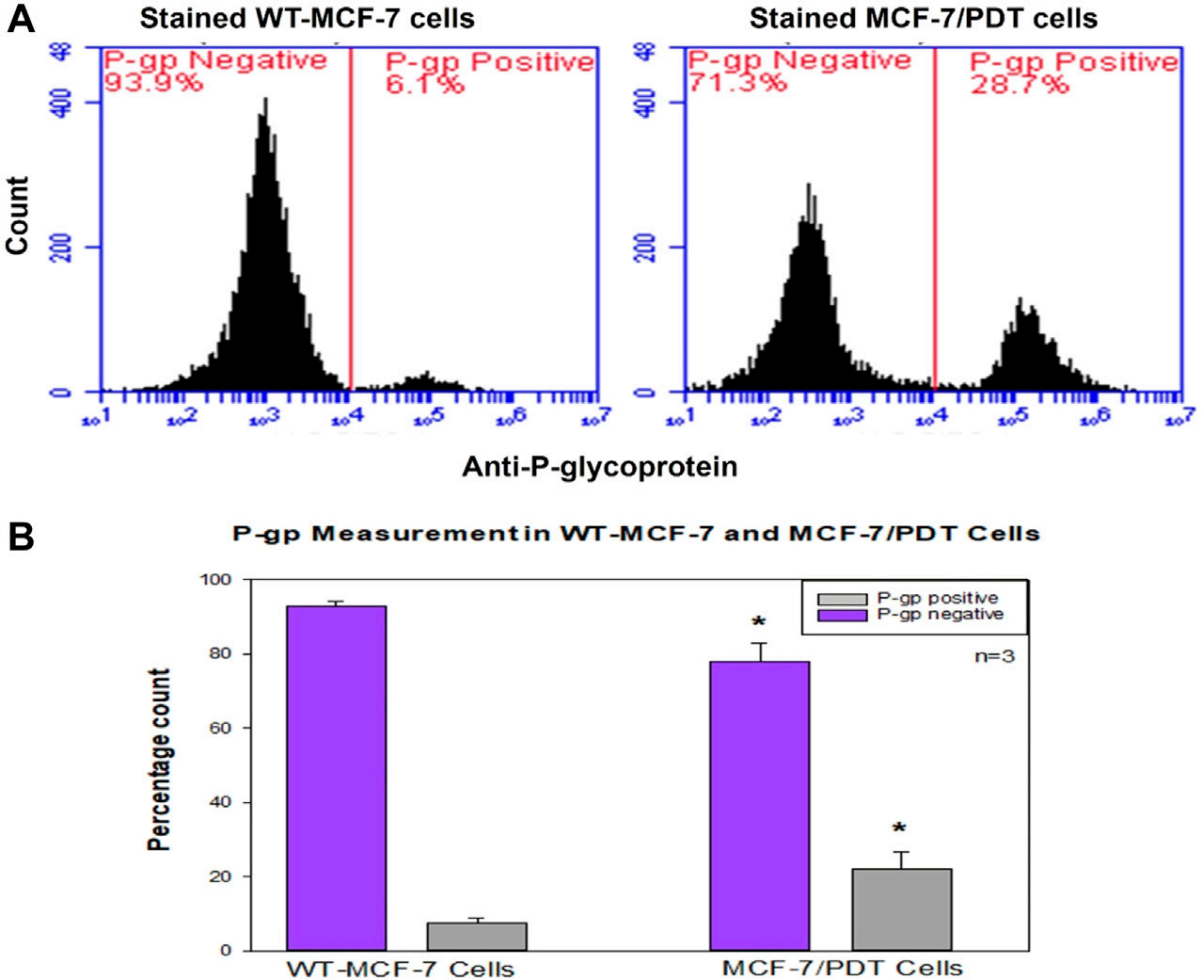
Flow cytometry measurement of P-glycoprotein expression in WT-and MCF-7/PDT cells. **(A)** Histogram expression of P-glycoprotein in both cells where MCF-7/PDT cells have a slight p-gp of 28.7% compared to 6.1% in WT-MCF-7 cells. **(B)** Bar chart representation of three independent repeats with significant difference (*p* = 0.040) of compared p-gp measurement in WT-and MCF-7/PDT cells.

Since the amount of P-gp is a significant hindrance to the PS accumulation and phototoxicity in the cell, we examined the expression of this protein in both the parental, 1st, 5th, and 10th generation cells by flow cytometry with monoclonal mouse P-gp antibody (MA5-13854) after 4 h of incubation and counterstained with FITC goat anti-mouse (NovusBio, NB720) for 1 h. The unstained control was used for negative control and did not detect the p-gp expression, whereas the differential percentage expression of P-gp on wild-type MCF-7 and cells from the 10th generation at 6.1% and 28.7%, respectively (Figure 2), was statistically different at *p* = 0.040.

### 3.3 Immunofluorescence detection of P-glycoprotein expression

**FIGURE 3:**
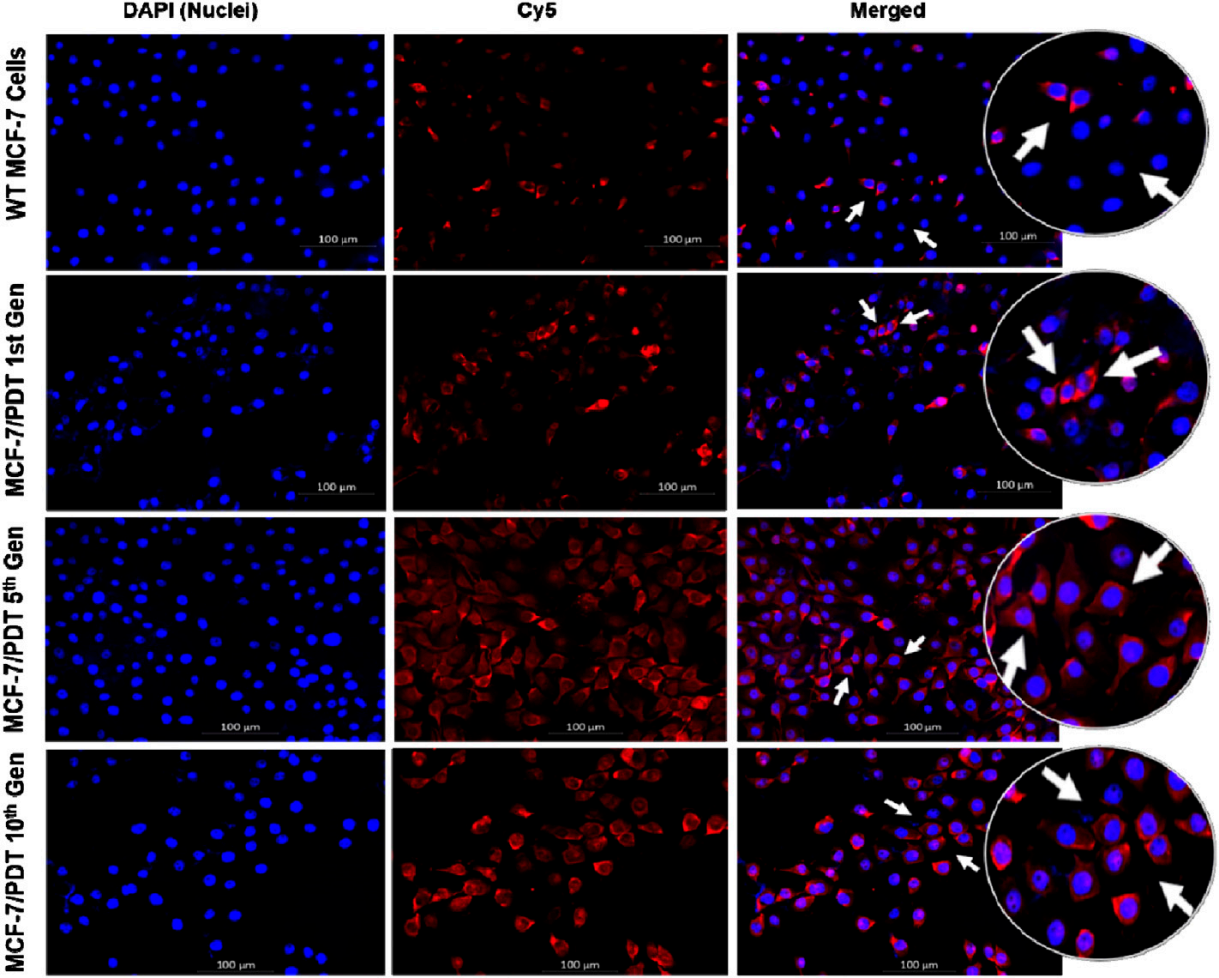
Immunofluorescence expression of P-gp in both WT and MCF-7/PDT cells. The cell nuclei were stained blue, and P-gp was stained with cy5 (red).

The expression of P-gp, one of the efflux proteins, was analyzed through indirect immunofluorescence. Similarly, monoclonal mouse P-gp antibody (MA5-13854) was used and counterstained with Cy5 (red) goat anti-mouse (NovusBio, NB7602) for 4 h. DAPI was also used to stain the nuclei. There was too little expression of P-gp in WT cells compared to the other generation cells. P-gp expressions were found in the membrane and cytoplasm of the cell population analyzed, which is considered a barrier to accumulating PS. These expressions were seen as highest in the 10th generation (Figure 3).

### 3.4 Measurement of superoxide dismutase enzyme activity

**FIGURE 4:**
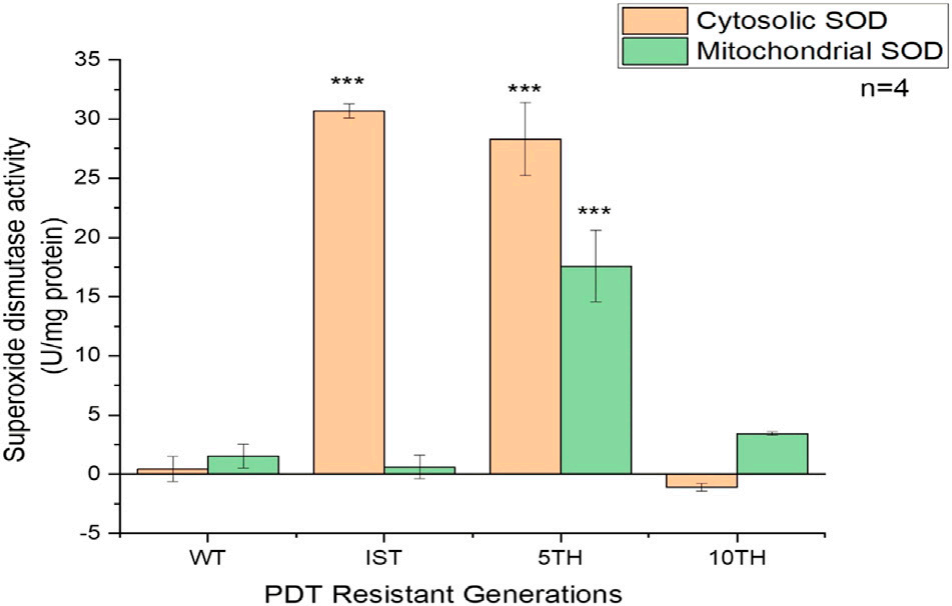
Superoxide dismutase activity in the cytosol and mitochondria of WT- and three generations of MCF-7/PDT cells were measured using the SOD activity assay kit. Increased enzyme activity was seen in the cytosol compared to the mitochondria. Significant differences were presented as *p < 0.05, **p < 0.01, and ***p < 0.001.

The assessment of the SOD enzyme was conducted to see its involvement in the development of resistant MCF-7 cells. The result showed an initial increased activity of the SOD enzyme in the cytosol of the 1st and 5th resistant cell generations compared to the mitochondrial SOD activity. Subsequently, at the 10th generation of resistant development, the activities of SOD were quenched and were only conserved in the mitochondria. Hence, its increased activity was observed (Figure 4).

### 3.5 Cell survival measurement of viability and proliferation

**FIGURE 5:**
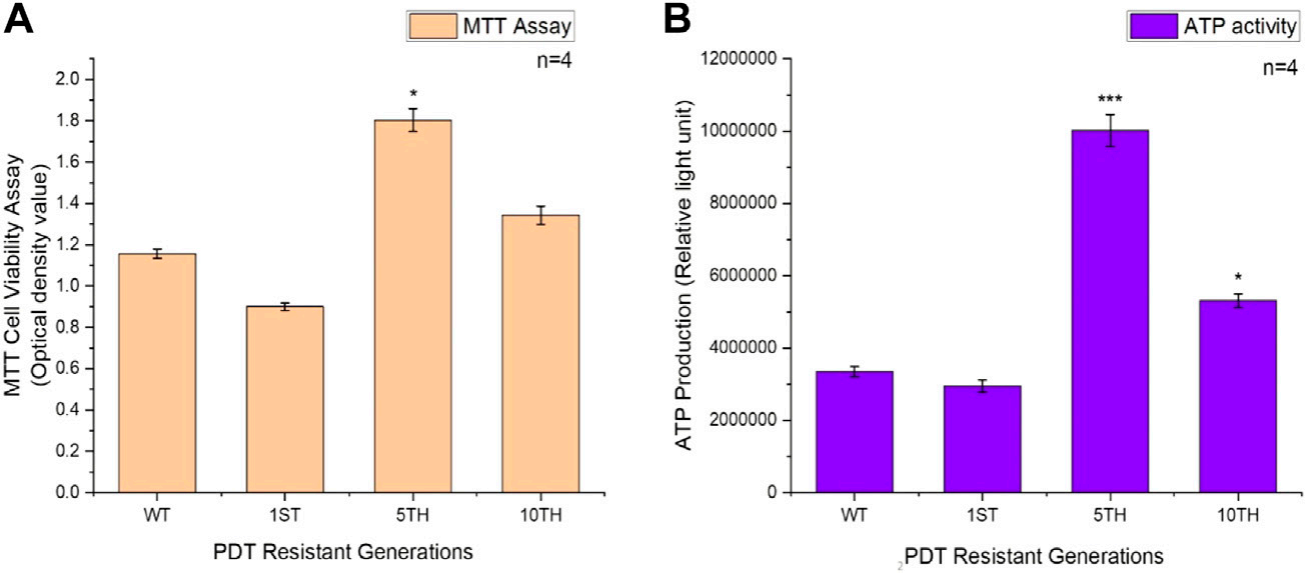
Represents the MTT and ATP cell growth assays of WT-MCF-7 and MCF-7/PDT cells. **(A)** An increase in the viability of the 5^th^ generation of MCF-7/PDT cells and **(B)** a significant increase in the ATP activity in MCF-7/PDT cells compared to the WT-MCF-7 cells. Significance differences as compared to WT-MCF-7 cells were presented as *p < 0.05, **p < 0.01, and ***p < 0.001.

We examined the cell viability of the resistant sublines of MCF-7 cells isolated after the 1st, 5th, and 10th resistant generations. MTT results revealed an increased significant difference in the viability of MCF-7/PDT 5th generation cells compared to the parental cells (Figure 5A). Similar results were obtained from the ATP metabolic activity. Although the latter measures the ATP content of the cells, we observed that significant (*p* < 0.001) amount of increased ATP content in the MCF-7/PDT 5th generation cells (Figure 5B).

### 3.6 Examination of cell cycle distribution

**FIGURE 6:**
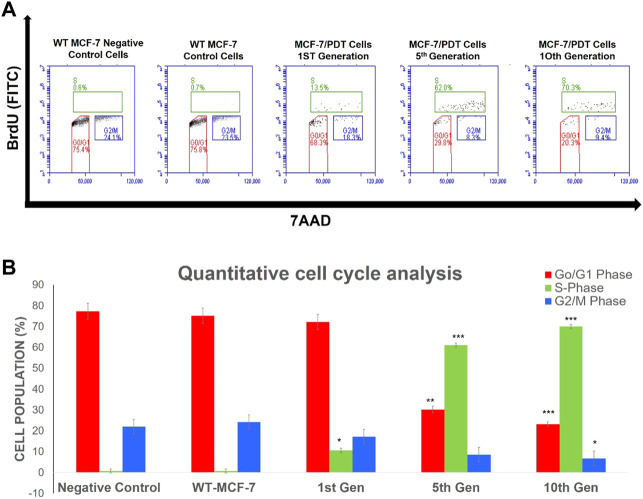
Percentage population of cells going through the cell cycle phases of proliferation (S), resting (G0/G_1_), and mitosis (G_2_/M) captured in different quadrants **(A)** by using 5-bromo-2-deoxyuridine (BrdU) and 7-amino-actinomycin D (7AAD). Quantitative analysis of the cell cycle event **(B)** showed a significant increase in the S-phase was noted in the generations of MCF-7/PDT cells. Significant differences as compared to WT-MCF-7 cells were presented as *p < 0.05, **p < 0.01, and ***p < 0.001.

Flow cytometric assessment of cell cycle distribution revealed that the proportion of cells in the G0/G1, S, and G2/M phases was 75.8, 0.7, and 23.5%, respectively, for parental cells; 63.8, 13.5, and 18.3%, respectively, for PDT first generation; 29.8, 62.0, and 8.3%, respectively, for 5th; and 20.3, 70.3, and 9.4%, respectively, for 10th PDT generations (Figure 6A). The parental cells and first-generation PDT resistant cells exhibited shorter S and G2/M phases with a concomitantly more extended G0/G1 phase in cell cycle distribution than the 5th generation (*p* < 0.01) and 10th generation (*p* < 0.001) (Figure 6B).

## 4 Discussion and conclusion

The PDT strategy involves localization of PS in organelles such as the mitochondria, lysosome, nucleus, and plasmatic membrane and subsequent illumination to activate a signaling cascade that leads to tumor cell death (Mroz et al., 2011). However, the emergence of resistant cells has shown to be a problem with PDT and other cancer therapies (Milla et al., 2011). The inability to induce apoptosis in response to PDT offers a selective advantage in tumor progression and, thus, resistance to therapy. This work aimed to isolate and perform an initial characterization of human MCF-7 cells resistant to photodynamic treatment. We used ZnPcS4 PS at 20 µm with 20 J/cm^2^ fluence laser irradiation over a repeated ten cycles of PDT to develop MCF-7/PDT cells and characterized these cells compared with their WT cells. The several PDT rounds allowed resistance induction and were similar to the methods used to obtain resistant PDT cells with ALA (Casas et al., 2006) and Photofrin PS (Singh et al., 2001). Our results using ZnPcS4-PDT showed that PDT conditions must be rigorous, intense, and prolonged to obtain resistant cells with higher cellular viability. The explanation for using protracted therapies like PDT to generate resistant cancer sublines could be the hypothesis that cancers are heterogeneous. Its treatment with PDT may perhaps positively select those with resistant strains that will withstand PDT cytotoxicity. This approach has previously been used to selectively isolate resistant cancer (Luna and Gomer, 1991).

The generated MCF-7/PDT cells morphologically showed a more fibroblastic and dendritic cell growth pattern with decreased doubling time than its WT MCF-7 cells (Figure 1). These features were stable over several passages during the experimentation time. These observations were similar to a study that uses ALA-PDT to develop resistant clones from the mammary adenocarcinoma LM3 line (Casas et al., 2006). Higher activity and expression of P-gp characterized the MCF-7/PDT cells grown with multiple cycles of PDT. P-gp plays a role in the resistant development of most breast cancers (Ge et al., 2017). This protein is a member of the efflux transporters that reduces the accumulation of cytotoxic compounds in the cells. The difference in the p-gp expression on WT and MCF-7/PDT cells could be responsible for increased efflux of PS that resulted in attenuated PDT efficacy. The flow cytometry and immunofluorescence analysis of p-gp expression (Figures 2A, 3), respectively, supported the increased expression of the p-gp protein, showing that MCF-7 resistant cells might have developed an enhanced protein matrix after repeated cycles of PDT. Reported evidence has supported the correlation of increased p-gp expression to homeostasis disruption of reactive oxygen species (Kuo, 2009). ROS formation is the principal mechanism of PDT. Thus, it could be deduced that elevated ROS formation of PDT modulated the transport mechanism at the epigenetic level, the increased expression of p-gp. Superoxide dismutase (SOD) forms the first line of defense against oxidative stress-induced cell damage produced by PDT (Dąbrowski, 2017). Notably, excess oxidative stress in cancer cells leads to increased reactive oxygen species (ROS) production, and SOD activity plays an essential physiological role in mitigating ROS’s harmful effects, leading to treatment resistance. The SOD assay results showed an initial cytoplasmic compared to the mitochondrial SOD activity. This antioxidant defense system is envisaged to increase its activity that chelates ROS and reduces the harmful effects of PDT, which may contribute to resistance development in MCF-7 cells. Other studies have reported a significant difference in metabolic pattern, intracellular ROS, and GSH ratio between sensitive and resistant-MCF-7 cells (Ge et al., 2017).

Cell population doubling time of WT and MCF-7/PDT cells was measured by direct counting of cells, and the doubling time of MCF-7/PDT cells was longer than that of WT cells. These were the first significant changes observed, and to understand which cell cycle phases were associated, cell cycle distribution was analyzed in WT and MCF-7/PDT cells. Cell cycle analysis showed an increased number of resistant cells at the S-phase, unlike the G0/G1 phase in its WT subline. The S-phase cells have increased the DNA synthesis, and studies on patients with reduced or unresponsive apoptosis in chemo- and radiotherapy-treated cells grow their DNA doubling efficiency and, thus, proliferate with multiple micronuclei (Driessens et al., 2003). We observed an increased cell population among the resistant cell generations at S-phase, which surmised that PDT damage to DNA that ordinarily could have led to apoptosis was metabolically being repaired at S-phase, unlike the untreated control WT cells. It is also notable that the MCF-7/PDT cells were very viable, but their doubling time was reduced compared to WT cells. Cell proliferation and viability were weaker in the 1st generation of resistant cells but increased at the 5th and 10th generations in relation to their WT cell counterparts. This could infer that a more significant percentage of cells in 1st generation have not yet recovered or still trying to adapt to the PDT effect. Cell cycle arrest increased DNA synthesis, and repair are some of the cell defense mechanisms against DNA damage and replication stress that prevent mutation formation, eventually leading to apoptosis (Jackson and Bartek, 2009). P-gp as a member of the multidrug-resistant (MDR) protein family, aids in transporting PS out of the cells thus, reduces PDT efficacy in resistant cells (Ishikawa et al., 2010). Another resistance mechanism reported in drug-resistant cells is the sequestration of drugs in acid vesicles which traps and prevents the drug from reaching its target site (Lou et al., 2006). Not all cells within a tumor are sensitive to therapy which introduces the thought of cancer stem cells in therapy resistance. Cancer stem cells are a distinct subpopulation with the potential for unlimited cell division (Arnold et al., 2020). They challenge the effectiveness of tumor treatment and determine the response of cells within the tumor. The cancer stem cell model may explain the genetic and phenotypical differences associated with therapy resistance in tumors, even within the exact tumor clone (Adorno-Cruz et al., 2015). Herein within this study, breast cancer cells were not characterized. In our results, we can conclude that the formation of MCF-7/PDT cells was due to the increased P-gp expression that prevented the accumulation of PS for effective PDT. This assertion and other contributing factors that aid cell resistance like cell-to-cell adhesion and migration capacities and epigenetic regulations could form the basis of future research. It is believed that employing assays like the scratch wound assay and measurement of vinculin levels, survivin, b1-integrins, and other genetic studies would be ideal for supporting and fully understanding the oncogenic activities of cell proliferation survival, and invasion in resistant cells.

In conclusion, we have successfully developed and characterized MCF-7/PDT cells, which featured increased P-gp expression in the S-phase of the cell cycle. The new MCF-7/PDT cells will help study the biological characteristics of recurrent breast cancer to elucidate possible related mechanisms and improve PDT treatment designs for cancer resistance.

## Data Availability

The raw data supporting the conclusions of this article will be made available by the authors, without undue reservation.
